# Are interferon-gamma release assays reliable to detect tuberculosis infection in patients with rheumatoid arthritis treated with Janus kinase inhibitors?

**DOI:** 10.1371/journal.pone.0275329

**Published:** 2022-09-28

**Authors:** Rossana Scrivo, Emanuele Molteni, Chiara Castellani, Alessio Altobelli, Cristiano Alessandri, Fulvia Ceccarelli, Manuela Di Franco, Roberta Priori, Valeria Riccieri, Antonio Sili Scavalli, Francesca Romana Spinelli, Claudio Maria Mastroianni, Fabrizio Conti

**Affiliations:** 1 Rheumatology Unit—Clinical Internal, Anesthesiological and Cardiovascular Sciences, Sapienza University of Rome, Rome, Italy; 2 Saint Camillus International University of Health Science, Rome, Italy; 3 Infectious Diseases—Department of Public Health and Infectious Diseases, Sapienza University of Rome, Rome, Italy; Universita degli Studi di Roma Tor Vergata, ITALY

## Abstract

**Background:**

Screening for latent tuberculosis infection is recommended in patients with rheumatoid arthritis (RA) starting Janus kinase inhibitors (Jaki). Interferon (IFN)-gamma release assays (IGRAs) are increasingly used for this purpose. Jaki tend to decrease the levels of IFNs, questioning the reliability of IGRAs during treatment with these drugs.

**Objectives:**

To compare the performance of QuantiFERON-TB Gold Plus (QFT-P) and QFT Gold In-tube (QFT-GIT) in RA patients treated with Jaki.

**Methods:**

RA patients underwent QFT-P and QFT-GIT at baseline (T0), and after 3 (T3) and 12 months (T12) of treatment with Jaki. The agreement between the two tests was calculated. The agreement between IGRAs and tuberculin skin test (TST) or chest radiography at baseline was also determined. The variability of QTF-P results was longitudinally assessed.

**Results:**

Twenty-nine RA patients (F/M 23/6; median age/IQR 63/15.5 years; median disease duration/IQR 174/216 months) were enrolled. A perfect agreement was found between QFT-P and QFT-GIT at all times (κ = 1). At T0, no agreement was recorded between IGRAs and TST (κ = -0.08) and between TST and chest radiography (κ = -0.07), a low agreement was found between QFT-P and chest radiography (κ = 0.17). A variation of 33.3% in the results of QFT-P was recorded at T3 vs T0, of 29.4% at T12 vs T0, and of 11.8% at T12 vs T3. The median levels of IFN-γ produced by lymphocytes in response to the mitogen of QFT-P decreased after 3 months followed by an increase after 12 months (not significant). No change in the median number of circulating lymphocytes was documented. Glucocorticoids intake was associated with a higher probability of negative or indeterminate IGRA results at T0 (p<0.0001).

**Conclusion:**

A response to IGRAs is detectable during treatment with Jaki. However, fluctuations in the results of IGRAs have been observed in the absence of correlation with clinical outcomes, thus challenging their interpretation.

## Introduction

In the last years, the therapeutic opportunities for patients with rheumatoid arthritis (RA) have been widened by the development of Janus kinase inhibitors (Jaki), including baricitinib and tofacitinib (O’Shea). These drugs are directed against the JAK/STAT (Janus Kinases/Signal Transducer and Activator of Transcription) pathway, which is responsible for the intracellular signal transduction of the type I and type II cytokine receptor family, acting as receptors of interferons (IFN), interleukins and colony-stimulating factors [1]. Because of their immunosuppressive action, screening for latent tuberculosis infection (LTBI) is recommended before starting the treatment [2]. Currently, IFN-γ release assays (IGRAs) and/or tuberculin skin test (TST) are used for this purpose, although they do not provide direct evidence of infection nor distinguish between latent and active disease [3]. Among IGRAs, QuantiFERON-TB Gold In-Tube (QFT-GIT) and the newer QuantiFERON-TB Gold Plus (QFT-P) version are largely used, and in patients with TB as a reference standard, they showed a sensitivity of approximately 70% for QFT-GIT and 90% for QFT-P, whereas that of TST was approximately 80% [4–6]. However, in immunocompromised patients, false-negative results in both TST and IGRAs can be observed [7–9]. Furthermore, dynamic changes in IGRA results have been reported in patients treated with biological agents [10–13], while the performance of these tests is not known during treatment with Jaki. Indeed, considering that Jaki modulate intracellular cascades, leading to a decrease in the levels of several immune mediators, including IFN-γ, a possible interference is suggestive. Therefore, the aims of this study were the following: 1) to assess the agreement between TST and chest radiography with both IGRAs in patients with RA screened for TB infection before starting Jaki 2) to assess the agreement between QFT-P and QFT-GIT in patients with RA screened for TB infection before starting Jaki 3) to assess the agreement between QFT-P and QFT-GIT in patients with RA during treatment with Jaki 4) to determine whether QFT-P and QFT-GIT present dynamic changes in patients with RA during treatment with Jaki.

## Materials and methods

The study received Policlinico Umberto I Ethics Committee approval in accordance with local requirements (prot. n. 674/18) and written informed consent was obtained from each participant.

A longitudinal, prospective study was carried out at Sapienza Arthritis Center outpatient clinic, Sapienza University of Rome, Rome, Italy, on patients with RA classified according to ACR/EULAR 2010 criteria [14] and candidates for treatment with baricitinib or tofacitinib. At enrolment (T0), demographic data, disease duration, past and current therapies, previous vaccination with BCG, history of TB contact, past diagnosis of active or latent TB and relative treatment were all registered on a digital database. All patients were required to undergo TST, chest radiography, QFT-P, and QFT-GIT.

TST was considered positive when the size of the reaction, determined by measurement of the induration, was ≥5 mm [15]. QFT-GIT is based on IFN-γ detection after stimulation by a combination of M. tuberculosis (Mtb) antigens in the presence of a positive (phytohemagglutinin–PHA) and negative control [16, 17]. The new generation IGRA, i.e. QFT-P, uses the same principle and procedures of QFT-GIT, but further includes TB1 and TB2 tubes in which a “CD4 only” or a “CD4 and CD8” response is elicited, respectively [18], enabling a more accurate assessment of cell-mediated immune response to TB infection [19].

Both QFT-P and QFT-GIT were repeated after 3 (T3) and 12 (T12) months since the onset of baricitinib or tofacitinib to evaluate their agreement and to assess possible dynamic changes in the results. QFT-P (Qiagen GmbH, Hilden, Germany) and QFT-GIT (Cellestis Limited, Carnegie, Victoria, Australia) were performed according to the manufacturer’s instructions.

Finally, at each time of observation, patients were asked to provide a blood sample for the evaluation of the number of circulating lymphocytes.

### Statistical analysis

Statistical analysis was performed by GraphPad Prism version 6 (GraphPad Software, San Diego, CA, USA). Data were expressed as median/IQR and percentages for continuous and categorical variables, respectively. The production of IFN-γ in response to antigenic stimulation was expressed in International Units/mL (IU/mL). To analyse concordance, Cohen’s κ coefficient (none <0.01, poor 0.01–0.20, fair 0.21–0.40, moderate 0.41–0.60, good 0.61–0.80, very good 0.81–1.00) was used after excluding indeterminate results for IGRA tests. Non-parametric tests of Mann-Whitney and Kruskal-Wallis test with Dunn’s post hoc test were used to analyze the differences between 2 or 3 groups, respectively, while comparisons in the different time points within subjects were studied using the Wilcoxon signed ranks. The comparison of percentages was performed using the χ2 test or Fisher’s exact test when appropriate. The significance of any correlation was determined by Spearman’s rank correlation coefficient. To assess the functional relationships among variables, linear regression was used. All differences were considered statistically significant when p<0.05.

## Results

The demographic and clinical features of our patients are reported in Table 1. Twenty-nine RA patients were consecutively enrolled (F/M 23/6; median age/IQR 63/16.5; median disease duration/IQR 174/126 months). Among them, 22 (75.9%) patients were to start baricitinib, and 7 (24.1%) tofacitinib.

**Table 1:** Clinical and demographic features of the patients enrolled (n = 29).

**F/M**	23/6
**Median age/IQR (years)**	63/16.5
**Median disease duration/IQR (months)**	174/126
**Rheumatoid factor (n/%)**	17/80.9
**anti-CCP antibodies (n/%)**	13/61.9
**Previous therapy with anti-TNF agents (n/%)**	21/72.4
**Treatment (n/%)**	
Methotrexate	11/37.9
Leflunomide	2/6.9
Sulfasalazine	3/10.3
Hydroxychloroquine	2/6.9
Glucocorticoids	22/75.9
DMARDs + Glucocorticoids	13/44.8
**No DMARDs (n/%)**	4/13.8
**Treatment dose (median dose; IQR)**	
Methotrexate (mg/week)	15; 5
Leflunomide (mg/day)	15; 10
Sulfasalazine (gr/day)	2; 2
Hydroxychloroquine (mg/day)	400; 0
Glucocorticoids (mg/day; prednisone equivalent)	5.63; 5

IQR: interquartile range; DMARDs: disease modifying anti-rheumatic drugs

There was no difference between baricitinib and tofacitinib groups in terms of disease duration (median/IQR 14/17 and 14.5/11.25 years, respectively), in the frequency and dosage of glucocorticoids (median/IQR 4/5 mg vs 4,5/3,25 mg, respectively), and in the use of csDMARDs (47% of patients under baricitinib and 25% under tofacitinib (p = 0.602).

One patient from an area with a medium-high incidence of TB was vaccinated with BCG. Another patient had had household contact with an individual affected by TB. Three patients (10.3%) presented chest radiography suggestive of TB infection. Four patients (13.8%) had a history of TB that was properly treated and 3 of them were previously treated with anti-TNF agents. Patients with evidence of TB infection based on any of IGRA, TST, or chest radiograph results were considered affected by LTBI after excluding active TB and received a 9-month course of isoniazid prophylaxis, which was already fully completed when they started treatment with JAKi.

### Performance of QFT-P, QFT-GIT, and TST at baseline

At baseline, QFT-P and QFT-GIT showed identical results, being positive in 6/29 patients (20.7%), negative in 18 (62.1%), and indeterminate in 5 (17.2%). Twenty-four patients (82.7%) had undergone TST. In the patient with a previous BCG vaccination, the test resulted positive, while both IGRAs were negative. The concordance between QFT-P and QFT-GIT was excellent (κ coefficient 1), whereas TST and IGRAs showed no concordance (κ coefficient -0.08; 95% IC -0.22/0.05).

The median values of INF-γ released in response to the antigenic stimulation in TB1 (0.0 IU/ml; IQR 0.09) and TB2 (0.01 IU/ml; IQR 0.20) tubes of QFT-P and those of QFT-GIT (0.0 IU/ml; IQR 0.17) were not significantly different (p = 0.58).

In the 3 patients with chest radiography suggestive of TB infection, IGRA tests were negative in one patient, positive in another patient, and indeterminate in the other one, while TST was negative in all of them. All 3 patients were taking glucocorticoids (median dose/IQR 10/12.5 mg/day prednisone equivalent), and 2 were also taking methotrexate (MTX). The concordance of IGRAs with chest radiography was poor (κ coefficient 0.17; 95% IC -0.28/0.63), while TST did not show any correlation with chest radiography (κ coefficient -0.07; 95% IC -0.17/0.03).

In the 4 patients with a history of TB infection, IGRAs were positive in 2 cases, negative in one, and indeterminate in another one, whereas TST was negative in all of them. Three patients were taking glucocorticoids (median dose/IQR 15/25 mg/day), and all of them were also taking a conventional disease-modifying anti-rheumatic drug (DMARD): 2 MTX, one sulfasalazine, the last MTX in association with hydroxychloroquine. A fair correlation was observed between a history of TB infection and IGRAs (κ coefficient 0.40 95%; IC -0.06/0.88), while no correlation was reported with TST (κ coefficient -0.06; 95% IC -0.15/0.03) (Table 2).

**Table 2:** Concordance between different screening methods for latent TB infection at baseline.

	TST	QFT-P	QFT-GIT	Chest radiograph	Previous TB infection
TST	-	None	None	None	None
		(κ -0.08)	(κ -0.08)	(κ -0.07)	(κ -0.06)
QFT-P	None	-	Very good	Poor	Fair
	(κ -0.08)		(κ 1)	(κ 0.17)	(κ 0.40)
QFT-GIT	None	Very good	-	Poor	Fair
	(κ -0.08)	(κ 1)		(κ 0.17)	(κ 0.40)
Chest radiograph	None	Poor	Poor	-	-
	(κ -0.07)	(κ 0.17)	(κ 0.17)		

TB: tuberculosis; TST: tuberculin skin test; QFT-P: QuantiFERON-TB Gold Plus; QFT-GIT: QuantiFERON-TB Gold In-Tube

**Fig 1:**
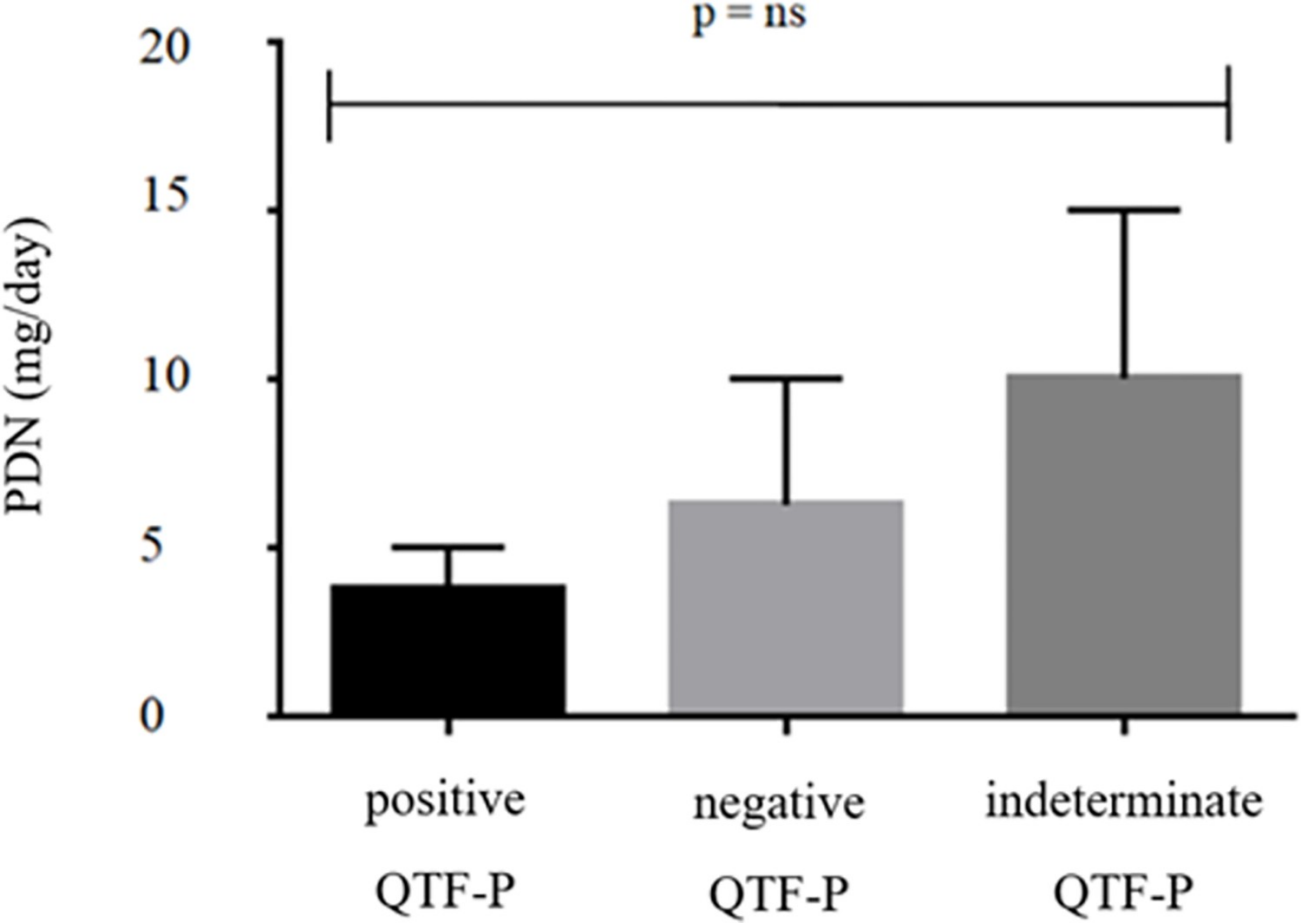
QFT-P results according to the dose of glucocorticoids. QFT-P: QuantiFERON-TB Gold Plus; PDN: prednisone.

The effect of therapy on the performance of the 3 tests was also evaluated. Patients with negative or indeterminate QFT-P and QFT-GIT results at baseline were more frequently taking glucocorticoids than those with positive results (83% vs 40%, p<0.0001), while no association with MTX or previous therapy with anti-TNF agents was observed. A tendency to more frequently develop negative or indeterminate QFT-P results with higher doses of glucocorticoids was also observed, however not in a statistically significant manner (p = 0.35) (Fig 1). In accordance, the correlation between the response to the PHA mitogen of QFT-P and the median dose of glucocorticoids (mg/day) showed a tendency to a reduced IFN-γ production (p = 0.33).

### Performance of QFT-P, QFT-GIT, and TST after 3 months of treatment with Jaki

Twenty-one patients out of 29 (72.4%) reached the 3-month follow-up of treatment with Jaki. Among them, QFT-P and QFT-GIT showed identical results, being positive in one case (4.8%), negative in 14 (66.7%), and indeterminate in 6 (28.6%) (κ coefficient 1). The comparison between the median values of IFN-γ produced in response to the antigenic stimulation in TB1 (0.0 IU/ml; IQR 0.04) and TB2 (0.01 IU/ml; IQR 0.05) tubes of QFT-P and those of QFT-GIT (0.0 IU/ml; IQR 0.02) did not show any significant difference (p = 0.53), confirming the very good concordance of the 2 tests.

**Fig 2:**
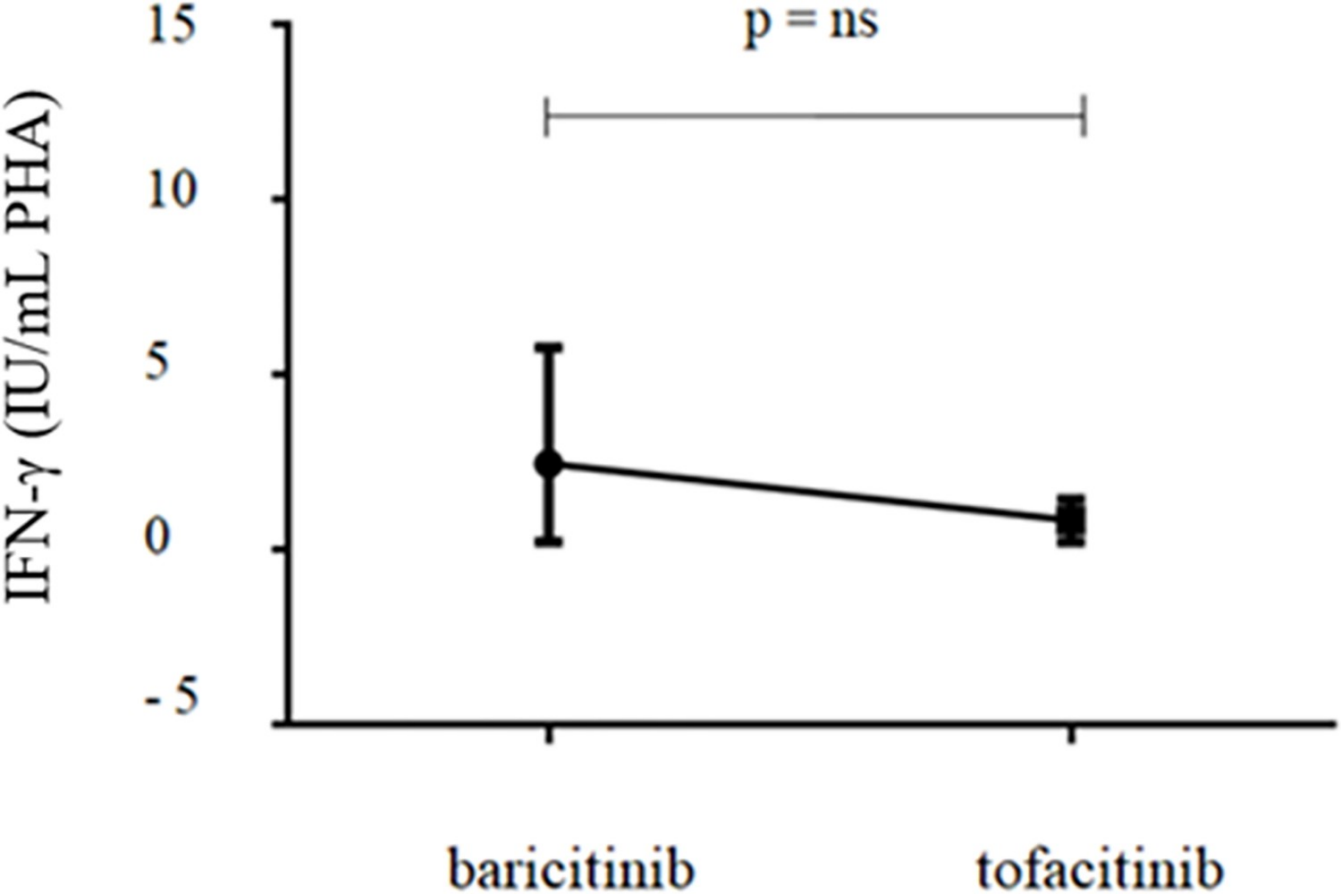
Median INF-γ values in response to PHA mitogen after 3 months of baricitinib and tofacitinib treatment. PHA: phytohemagglutinin.

The IFN-γ production to PHA mitogen was not different among patients treated with baricitinib than those treated with tofacitinib (p = 0.16), although in this last group lower levels were observed (Fig 2).

Of the 21 patients, 12 (57%) were treated with glucocorticoids at a median dose/IQR of 2/5 mg/day with a significant reduction compared to the baseline (median dose/IQR 5.63/5 mg/day; p = 0.02). No significant variation of INF-γ production in response to PHA mitogen of QFT-P was observed in those taking glucocorticoids.

### Performance of QFT-P, QFT-GIT, and TST after 12 months of treatment with Jaki

Seventeen patients out of 21 (80.9%) reached the 12-month follow-up of therapy with Jaki. Among them, QFT-P and QFT-GIT showed identical results, being positive in none, negative in 14 individuals (82.4%), and indeterminate in 3 (17.6%) (κ coefficient 1). The comparison between the median values of IFN-γ produced in response to the antigen stimulation in TB1 (0.0 IU/ml; IQR 0.05) and TB2 (0.03 IU/ml; IQR 0.06) tubes of QFT-P and those of QFT-GIT (0.01 IU/ml; IQR 0.05) did not produce any significant difference (p = 0.74), confirming the excellent concordance of the 2 tests even at 12 months of therapy with Jaki.

In patients with a positive QFT-P test at baseline, no difference in the median/IQR levels of IFN-γ produced in response to the TB1 antigens (0.63/3.11 IU/ml) and those produced in response to the antigens included in the TB2 tube (0.62/3.04 IU/ml) was observed. Likewise, no significant difference was found comparing the median levels/IQR of IFN-γ in the TB1 (0.37/7.24 IU/ml) and TB2 (0.40/7.70 IU/ml) of the 4 patients with a previous diagnosis of TB infection.

### IGRA variability during treatment with Jaki

IGRA results at the 3 times of observation are summarised in Table 3. Of the 6 patients who tested positive before the start of Jaki, 5 reached the follow-up at 3 months. At this time point, a positive result was confirmed in only one case, a reversion from a positive to a negative result was observed in 2 patients, while the other 2 showed an indeterminate result.

**Table 3:** IGRA results at baseline and after 3 and 12 months of treatment with Jaki.

	*T0*	*T3*	*T12*
	*n = 29*	*n = 21*	*n = 17*
***Positive*, *n (%)***	6 (20.7)	1 (4.8)	0 (0)
***Negative*, *n (%)***	18 (62.1)	14 (66.7)	14 (82.4)
***Indeterminate*, *n (%)***	5 (17.2)	6 (28.5)	3 (17.6)

IGRA: Interferon-gamma release assays; Jaki: Janus kinase inhibitors

At 12 months, IGRAs showed a reversion in the sole patient positive at T3, the 2 negative patients remained negative, while of the 2 indeterminate patients at T3, one discontinued Jaki before 12 months and the other became negative.

Of the 5 patients with indeterminate IGRAs before the beginning of the therapy with Jaki, 3 reached the follow-up at 3 months. Among them, 2 remained indeterminate at 3 and 12 months, while the other had a negative result in all the times of observation.

Of the 18 IGRA-negative patients at baseline, 13 reached the follow-up at 3 months and 10 at 12 months. At T3, IGRAs turned indeterminate in 2 cases and were confirmed negative in one of the 2 patients at T12, while the other was lost at follow-up. No cases of conversion from negative to positive were observed in the follow-up.

Variability of the results of IGRAs was observed in 33.3% of cases at T3 and 29.4% at T12 compared to baseline, and in 1.8% at T12 compared to T3. Therefore, the widest variety of the results was observed after 3 months of therapy with Jaki.

**Fig 3:**
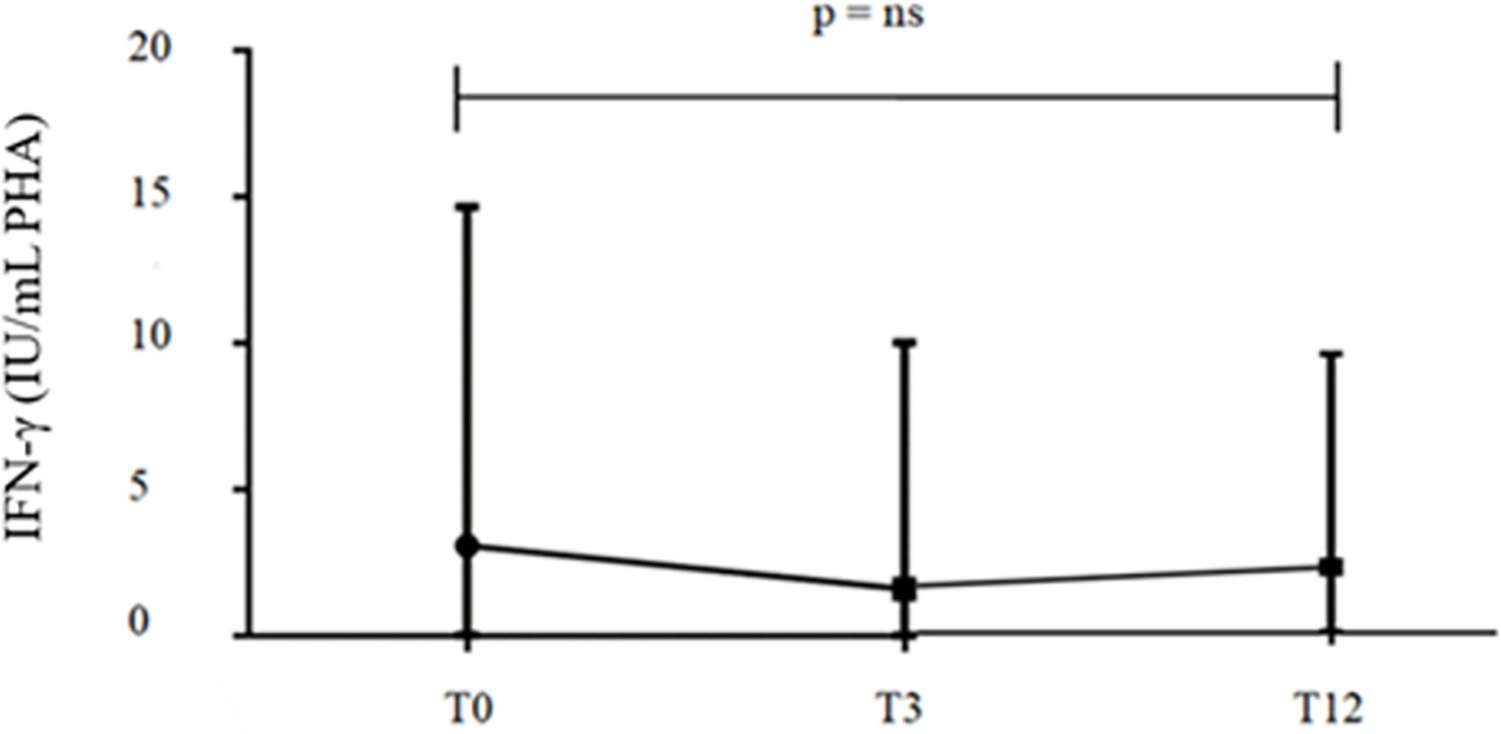
Comparison between the median values of INF-γ in response to the PHA mitogen of QFT-P before and after 3 and 12 months of therapy with Jaki. PHA: phytohemagglutinin; QFT-P: QuantiFERON-TB Gold Plus; Jaki: Janus kinase inhibitors.

We also assessed whether IFN-γ production might be affected by Jaki. Median values of IFN-γ released in response to PHA in QFT-P before and after 3 and 12 months of Jaki therapy were 3.08/7.68 IU/ml, 1.59/4.72 IU/ml, and 2.25/4.61 IU/ml, respectively (Fig 3).

Finally, to understand whether the reduction of the lymphocytic count induced by Jaki could affect the result of the test, the median/IQR lymphocyte count of positive patients was calculated, which was inferior after 3 months (1815/690/mm^3^) compared to baseline (2140/750/mm^3^), although the difference was not statistically significant (p = 0.41).

## Discussion

IGRAs were developed to overcome the limits of TST in the diagnosis of TB infection. However, when serially repeated, the interpretation of their results may be complicated by the lack of an optimal cut-off and by the difficulty to explain dynamic changes [20], that were observed both in healthy individuals and in patients treated with biological agents [10–13]. No data are available in patients treated with Jaki, known to modulate intracellular cascades leading to a decreased production of several cytokines, including IFN-γ. Hence, because the interference of Jaki on IGRA performance is suggestive, we carried out this prospective study in patients with RA before and during Jaki treatment.

First, we showed the lack of concordance between TST and both the IGRAs tested, QFT-P and QFT-GIT. TST was negative in all patients, except in the BCG-vaccinated individual, in whom IGRAs were negative. In addition, 3 chest radiographs were suggestive of LTBI, but TST results were negative in all of them, while IGRAs were positive in 33.3% of the cases, showing a better performance compared to TST. Also in patients with previous TB infection TST seemed to be less reliable, being negative in 100% of cases versus 50% of IGRAs. All patients except one were taking glucocorticoids when they underwent TST and IGRAs. Although glucocorticoid assumption was more likely to be significantly associated with negative or indeterminate IGRA results, these tests appeared to be more sensitive than TST in immunosuppressed patients [21]. Conversely, MTX did not significantly affect IGRA results.

In the second place, we found that Jaki do not influence IFN-γ production. We observed similar levels of IFN-γ released in response to antigens included in the TB1 and TB2 tubes of QFT-P and in the test tube of QFT-GIT. The tube of QFT-GIT differs from TB1 of QFT-P in the antigens (TB7.7, apart from ESAT-6 and CFP-10 that are present also in TB1) and for their distribution inside the tube [22]. TB2 of QFT-P was introduced to increase the sensitivity of the test in individuals with recent contact with Mtb, in immunocompromised hosts, and in children, other than constituting a valid tool to distinguish patients with an active infection from those with an LTBI [22]. Even analyzing the group of patients with positive IGRAs, the comparison of IFN-γ produced by lymphocytes in response to TB1 antigens and those of TB2 showed no significant difference. Hence, TB2 did not add further accuracy in the setting of immunocompromised patients with RA. Furthermore, a perfect agreement between QFT-P and QFT-GIT was found in all observations, confirming published data [23, 24].

The variability of QFT-P during Jaki treatment was evaluated, showing the greatest changes at 3 months of therapy with Jaki. In particular, the variability was observed in 33.3% of cases at T3 and 29.4% at T12 compared to baseline, and in 1.8% at T12 compared to T3. To interpret these changes and analyze the hypothetical influence of the therapy with Jaki, we analyzed the IFN-γ levels of lymphocytes exposed to the PHA mitogen. After 3 months of therapy, we observed a reduction of these levels and an increase after 12 months, although these variations were not significant. The hypothesis that Jaki could alter the result of the test by reducing the number of circulating lymphocytes was investigated by comparing the lymphocyte count in positive patients at baseline, which later showed reversion, with those at T3. However, after 3 months of therapy, we observed a not significant reduction in the lymphocytic count.

Hence, both QFT-P and QFT-GIT were subject to variations of their results during Jaki therapy, although there is no proof that they may be solely due to the mechanism of action of these drugs. Yet, it was observed in 80% of positive patients at baseline, who had a reversion of the test result at 12 months of therapy. The negative results at T12 are likely to be due to immunosuppression, since the isoniazid prophylaxis course was already fully completed when patients started treatment with JAKi. Besides, prophylactic isoniazid had no effect on changes in QFT-GIT readouts in adults with positive TST in a high TB incidence setting [25].

Our study has some limitations. The first is represented by the small number of participants, which restrains the interpretation of the results, especially in terms of concomitant treatments, canceling the possibility to analyze separately the effects of glucocorticoids and MTX on IGRA results. Likewise, the unbalanced number of patients treated with baricitinib and tofacitinib does not allow speculation on the differences observed in the IFN-γ production in the two groups. Also, a throughout study on CD4 and CD8 lymphocytic number and function under different treatments was not performed.

Larger studies over greater periods should be conducted in the future to analyze the effect of isoniazid prophylaxis and immunosuppressants on IGRA outcomes and optimize the clinical use of these tests.

## Conclusion

In conclusion, we provide evidence of the feasibility of IGRAs in patients treated with Jaki despite their potential to suppress the production of IFN-γ. QFT-P and QFT-GIT showed excellent agreement. However, they also presented dynamic changes when serially repeated, not associated with the clinical conditions of the patients. These variations, also observed in patients treated with biological agents, hardly ease the interpretation of the results. A careful clinical judgment is still required to address treatment decisions in immunocompromised patients undergoing serial IGRAs.

## Supporting information

S1 File (XLSX)
